# Investigation of WO_3_ Electrodeposition Leading to Nanostructured Thin Films

**DOI:** 10.3390/nano10081493

**Published:** 2020-07-30

**Authors:** G. Mineo, F. Ruffino, S. Mirabella, E. Bruno

**Affiliations:** Dipartimento di Fisica e Astronomia “Ettore Majorana”,Università di Catania, and CNR-IMM, via S. Sofia 64, 95123 Catania, Italy; francesco.ruffino@dfa.unict.it (F.R.); salvo.mirabella@dfa.unict.it (S.M.); elena.bruno@dfa.unict.it (E.B.)

**Keywords:** tungsten oxide, electrodeposition, nanograins, nucleation, growth model

## Abstract

Nanostructured WO_3_ represents a promising material for electrochromic and sensing devices. In this scenario, electrodeposition is a promising low-cost approach for careful production. The electrodeposition of tungsten oxide film from a peroxo-tungstic-acid (PTA) solution is investigated. WO_3_ is synthetized onto Indium doped Tin Oxide (ITO) substrates, in a variety of shapes, from a fragmentary, thin layer up to a thick continuous film. Samples were investigated by scanning electron (SEM) and atomic force microscopy (AFM), Rutherford backscattering spectrometry (RBS), X-ray Diffraction analysis (XRD), energy gap measurement. Electrodeposition current curves are compared with characterization results to model the growth process. Early stages of electrodeposition are characterized by a transient cathodic current revealing an instantaneous nucleation followed by a diffusion limited process. A quantitative analysis of W deposition rate and current at working electrode validates a microscopic model for WO_3_ electrodeposition driving the process towards nanostructured versus continuous WO_3_ film.

## 1. Introduction

WO_3_ is an *n*-type semiconductor successfully used for many applications, such as in electrochromic [1,2,3] and sensing devices [4,5,6], thanks to its excellent chromic properties, inherent electrical conductivity and excellent sensitivity and selectivity toward CO [4], NO_2_ [5], NH_3_ [7], H_2_S [8] gases. Recently, nanostructured WO_3_ gained large scientific interest because of improved performances, with respect to bulk material [9,10,11,12,13,14]. In this scenario, the development of facile and repeatable synthesis of nanostructured WO_3_ can be an effective tool for exploitation of this interesting semiconductor as an active sensing material.

There are many techniques to synthesize nanostructured WO_3_: sputtering [15], thermal evaporation [16], sol-gel [17], electrodeposition [18] and hydrothermal [19]. Among them, the electrodeposition represents a promising approach since it combines low temperature, high control, and low-cost [20,21,22,23,24,25]. Typically, the used electrolyte is a peroxo-tungstic-acid (PTA) solution, which produces WO_3_ by an electroreduction process in acidic conditions. A long electrodeposition process leads to cracked WO_3_ films; still, the early stage of synthesis is disregarded in the literature despite the great potentialities in producing nanostructures. Understanding the growth kinetics at early stages is of paramount importance for WO_3_ nanostructure synthesis by electrodeposition. Pongoddi et al. reported on the electrodeposition of WO_3_ nanostructured thin films for gas sensing, by using a seed layer made by spin coating a WO_3_ sol [20]. In this case, the role of electrodeposition in the nucleation process cannot be extracted as the seed layer plays a crucial role. Kwong et al. studied WO_3_ films (thicker than 250 nm) prepared by electrodeposition with an aqueous solution of peroxotungstic acid at varying tungsten concentrations [25]. Even if a detailed investigation of the early stage of electrodeposition is missing, they concluded that the growth kinetics of the films start with an impingement/percolation of WO_3_ grains (80 nm in size). A detailed investigation of early stages during WO_3_ electrodeposition, with short times and cathodic potential variation, is truly missing despite that it can be extremely useful for nanostructures synthesis.

In this paper the electrodeposition process is quantitatively investigated, particularly at the early stages, for a careful synthesis of WO_3_ thin films. The electrodeposition potential and times are largely spanned, and morphological and compositional analyses were conducted to model the WO_3_ growth kinetics at early stages, which are the most interesting ones for WO_3_ nanostructures synthesis control.

## 2. Materials and Methods

### 2.1. Chemicals

**Figure 1:**
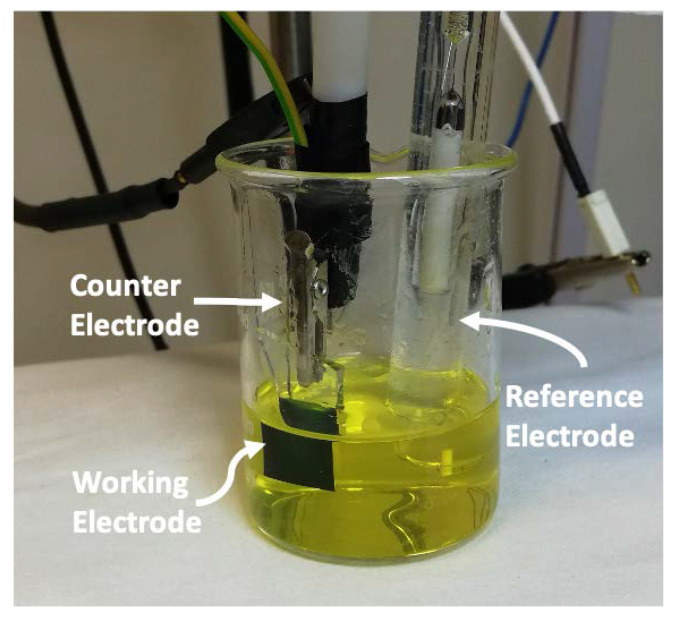
Experimental setup for the electrodeposition of WO_3_ films. It is possible to distinguish the working electrode, the counter electrode and the reference electrode. They are immersed into the peroxo-tungstic acid (PTA) solution.

Samples were synthesized by using an electrodeposition technique with a peroxo-tungstic-acid (PTA) solution (6.5 g of W metallic powder in a mixture of 40 mL of H_2_O_2_ (30%) and 4 mL of deionized water) [18]. As this reaction is exothermic, the PTA solution was constantly stirred for 24 h in a cold bath (between 0–10 °C). The obtained colorless solution was filtered with a 0.1 μm filter and refluxed for 6 h at 55 °C in order to remove H_2_O_2_ in excess. Then, dilution with an equal volume of anhydrous absolute ethanol was operated to prevent precipitation of peroxo complexes [10], yielding a yellow-colored PTA solution (Figure 1) with a pH of 1.24. Once cooled at room temperature, the solution was used as electrolyte during electrodeposition in a three-electrode electrochemical cell (Figure 1), with an Ag/AgCl electrode as the reference electrode, a platinum wire as the counter electrode, and an Indium-Tin-Oxide (ITO) coated glass as the working electrode. The ITO substrates were cut in 1 cm × 2 cm pieces and then washed with deionized water. An area of 1 × 1 cm^2^ was immersed in the PTA solution. Immersed area of each sample was measured after the process, in order to have comparable current density values. To prevent PTA degradation, the solution was stored at 4 °C between different synthesis and discarded one week after preparation.

Films were deposited under potentiostatic condition, by varying deposition time (t_d_) and applied potential (−V_d_) for different samples. Just after electrodeposition, the samples, promptly rinsed with deionized water, were dark blue, but fully bleached in 1 day at room atmosphere.

### 2.2. Characterization

The WO_3_ electrodeposition was carried out by using a Versastat 4 potentiostat (Ametek, Berwyn, PA, USA) in a three-electrode setup (Figure 1). All measurements were performed at room temperature and atmospheric pressure. The morphological analyses were carried out by a scanning electron microscope (SEM) Gemini Field Emission SEM Carl Zeiss SUPRATM 25 (FEG-SEM, Carl Zeiss Microscopy GmbH, Jena, Germany). The transmittance spectra were obtained by using a UV-VIS-NIR spectrophotometer Varian Cary 500 (Varian Inc., Palo Alto, CA, USA). Atomic Force Microscopy (AFM) studies were carried out by a Bruker-Innova microscope (Bruker Corporation, Billerica, MA, USA) operating in contact mode and employing ultra-sharpened Si tips (MSNL−10 from Bruker, with anisotropic geometry, radius of curvature ∼2 nm, tip height ∼2.5 μm, front angle ∼15°, back angle ∼25°, side angle 22.5°). The Si tips were substituted as soon as a resolution loose was observed during the AFM images acquisition. Each AFM image was acquired using a scan rate of 0.3 Hz and acquiring 512 × 512 lines. The acquired AFM images were analyzed using the SPMLABANALYSES V7.00 software. The WO_3_ films were also analyzed by X-ray diffraction using a Bruker Discover^TM^ diffractometer (Bruker Corporation, Billerica, MA, USA) equipped with thin film attachments and a K α Cu source. For the determination of W and O content, Rutherford backscattering spectrometry (RBS, 2.0 MeV He^+^ beam at normal incidence) with a 165° backscattering angle was employed, by using a 3.5 MV HVEE Singletron accelerator (High Voltage Engineering Europa, The Netherlands). RBS spectra were analyzed using XRump software (Thompson M., Windows (95-Win7)-Genplot and RUMP, Available online, http://www.genplot.com/download.htm).

## 3. Results and Discussion

### 3.1. Morphological Analyses

**Figure 2:**
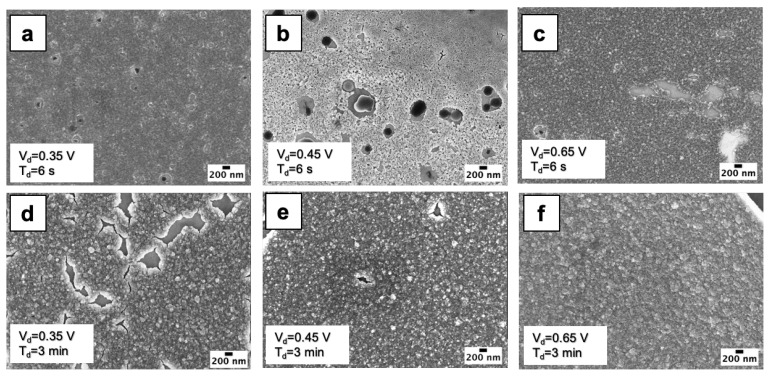
SEM images of electrodeposited WO_3_ by using: (**a**) V_d_ = 0.35 V and t_d_ = 6 s; (**b**) V_d_ = 0.45 V and t_d_ = 6 s; (**c**) V_d_ = 0.65 V and t_d_ = 6 s; (**d**) V_d_ = 0.35 V and t_d_ = 3 min; (**e**) V_d_ = 0.45 V and t_d_ = 3 min; (**f**) V_d_ = 0.65 V and t_d_ = 3 min. It is possible to observe the characteristic pattern with nanograins (50 nm lateral size) regardless of V_d_ and t_d_.

Figure 2 shows high magnification SEM images of electrodeposited films, obtained by using different electrodeposition parameters: V_d_ = 0.35 V and t_d_ = 6 s (a), V_d_ = 0.45 V and t_d_ = 6 s (b), V_d_ = 0.65 V and t_d_ = 6 s (c), V_d_ = 0.35 V and t_d_ = 3 min (d), V_d_ = 0.45 V and t_d_ = 3 min (e), V_d_ = 0.65 V and t_d_ = 3 min (f). By observing the high magnification SEM images (Figure 2a–f) a specific common pattern can be seen, revealing that WO_3_ film is made of nanograins with an average lateral size of 50 nm, regardless of V_d_ and t_d_. Figure S1 shows the AFM image of the sample deposited at V_d_ = 0.45 V for 6 s and the relative AFM line profile. The obtained size distribution confirms that the height of these nanograins is around 50 nm. Joining the AFM and SEM data we can assume that WO_3_ film is composed of 50 nm sized nanograins.

**Figure 3:**
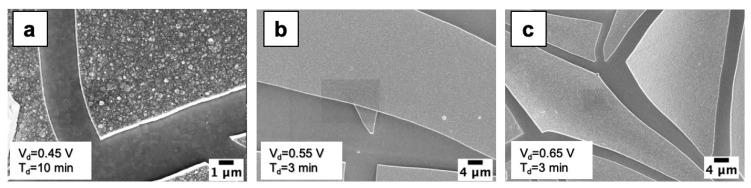
SEM images of electrodeposited WO_3_ by using: (**a**) V_d_ = 0.45 V and t_d_ = 10 min; (**b**) V_d_ = 0.55 V and t_d_ = 3 min; (**c**) V_d_ = 0.65 V and t_d_ = 3 min.

Figure 3a–c show SEM images of samples with broken WO_3_ films, with clear µm sized islands. The electrodeposition parameters used for these samples are the following: (a) V_d_ = 0.45 V and t_d_ = 10 min, (b) V_d_ = 0.55 V and t_d_ = 3 min and (c) V_d_ = 0.65 V and t_d_ = 3 min. The film breaking is probably caused by high mechanical stress, due to lattice mismatch, for thick WO_3_ film [14,26]. Thus, while for low V_d_ and t_d_ the films are continuous (e.g., V_d_ = 0.45 V for t_d_ < 10 min or V_d_ = 0.65 V for t_d_ < 3 min), increasing V_d_ and/or t_d_ leads to cracked films, as visible at V_d_ = 0.45 V t_d_ = 10 min (Figure 3a) or at V_d_ = 0.55 V t_d_ = 3 min, as well as at V_d_ = 0.65 V t_d_ = 3 min (Figure 3c). The WO_3_ islands formed after the film cracking possess the same pattern (Figure 2f), meaning that also for longer electrodeposition times WO_3_ nanograins are present.

**Figure 4:**
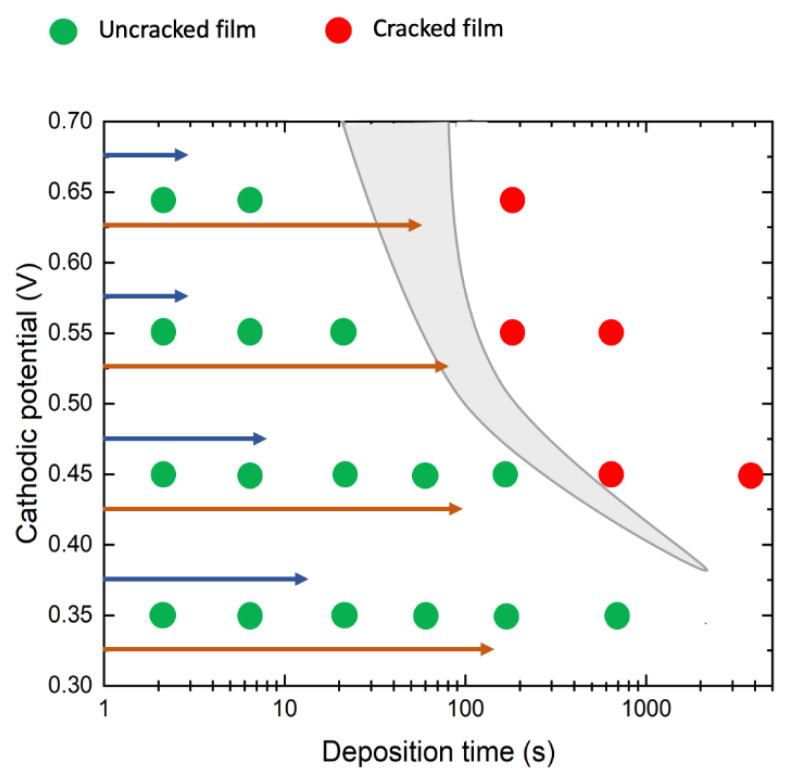
Deposition map: each electrodeposited sample is reported here as a function of V_d_ and t_d_. The WO_3_ films are reported as green or red dots if they result uncracked or cracked, respectively. Per each deposition at fixed V_d_, two arrows indicate the time extent to get the maximum current (t_m_, blue arrow) or the saturation current (t_s_, orange arrow) measured during the electrodeposition (see Figure 5).

For investigating the early stage growth kinetics, an uncracked film deposition is needed, thus it was useful to correlate electrodeposition parameters (V_d_ and t_d_) with occurrence of film cracking. In order to do this, all samples were analyzed by SEM and placed in a deposition map (Figure 4) as a function of V_d_ and t_d_. The uncracked (cracked) films are reported with green (red) balls. It is possible to individuate a border (grey colored region) between cracked and uncracked films, allowing to choose the parameter combination leading to desired WO_3_ films. As expected, the higher the potential is, the shorter the time to get uncracked films. In literature, to investigate the electrodeposition of WO_3_ films, a cathodic potential of 0.45 V for 10 min is often used [26,27]. From Figure 4 we observe that these parameters lead to cracked film, even if this combination lies very close to the border. In Figure 3a we show that using V_d_ = 0.45 V and t_d_ = 10 min leads to quite large (tens of µm) islands.

Even if small differences in concentration of PTA solution and/or in lab procedures can affect the border line position in our deposition map, we preferred to stay well apart from that border in order to proceed with a quantitative investigation of growth kinetics of WO_3_ film. Thus, in the following, only uncracked samples will be considered.

Figure S2 shows the Tauc plot obtained from a transmittance spectrum of a sample deposited with V_d_ = 0.45 V for 3 min. Such a combination of parameters allows to have a WO_3_ film thick enough to create a homogeneous film, but uncracked (Figure 4). The linear fit (red line) to Tauc plot gives information about the band gap [28], which results in 3.37 ± 0.01 eV. No correlation between the band gap value and the electrodeposition parameters is found, since the measured optical band gap in several samples ranges between 3.35 and 3.38 eV, regardless of the electrodeposition parameters. This value suggests that the film is amorphous, according to the data reported in the literature [10]. The XRD pattern of this film is reported in Figure S3, showing only peaks related to the ITO substrate and confirming the amorphous phase of the electrodeposited WO_3_ films.

### 3.2. Nucleation and Growth of WO_3_ Films

**Figure 5:**
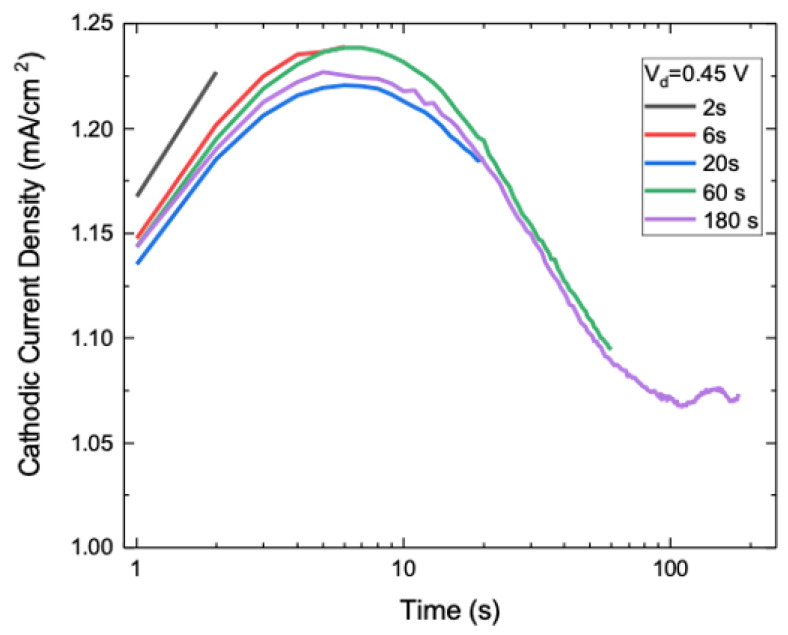
Current transients recorded during the samples electrodeposition at V_d_ = 0.45 V.

During electrodeposition, the cathodic (negative) current is acquired as a function of time, giving insights into the kinetics of the process. As an example, in Figure 5 (lin-log scale) we report the transient of cathodic current density for five samples obtained at V_d_ = 0.45 V, by varying t_d_ from 2 s to 180 s. The same current transient is showed by all samples, as expected, with an increase up to a maximum (I_m_) at 7 s (t_m_) and a decrease leading to a plateau after around 100 s (t_s_). The measured value for current density is quite in agreement with those reported by Kwong et al. [25] at similar cathodic potential, still in our case a clear transient with a bell shape is observed.

During the first 100 s, the current shows a variation of more than 20% in its value and such a feature tells about early stage kinetics of the electrodeposition process. It should be noted that similar trends also occur for other cathodic potentials (Figure S4) with a time extent of transient lower at higher cathodic potential.

In order to account for the current transient, we considered the Sharifker–Hills model (S-H model) [29], which describes the film growth process during electrodeposition in terms of nucleation and diffusion processes. According to the S-H model, nucleation can be instantaneous or progressive. In the instantaneous case, all nucleation centers are speedily formed as electrodeposition starts and nuclei density remains constant from there on. A diffusive region starts to grow up around each nucleus leading to current increase; as soon as diffusion regions overlap each other, at t = t_m_, a current decrease occurs, because of the transition from convergent to linear diffusion. In the progressive nucleation, nuclei are continuously formed during growth, so that nuclei density increases linearly with time. Also in this case, a transition from convergent to linear regime is obtained at t = t_m_.

**Figure 6:**
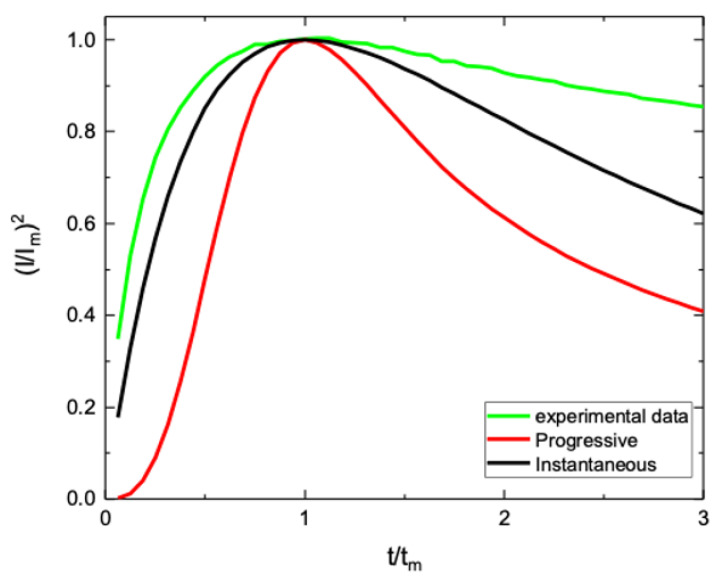
Comparison between the experimental data (green curve) and the theoretical data of the Sharifker–Hills model for instantaneous and progressive nucleation (black and red curve, respectively). The experimental curve is related to electrodeposition of WO_3_ by using V_d_ = 0.35 V and t_d_ = 3 min.

In Figure 6, kinetics transients for instantaneous and progressive nucleations, as described by the S-H model, are reported as normalized current, according to the following relation:

(1) $$\frac{I^{2}}{I_{m}^{2}}=\frac{1.9542}{t/t_{m}}{\left\{1-\exp\left[-1.2564\left(t/t_{m}\right)\right]\right\}}^{2}$$

for instantaneous nucleation and

(2) $$\frac{I^{2}}{I_{m}^{2}}=\frac{1.2254}{t/t_{m}}{\left\{1-\exp\left[-2.3367{\left(t/t_{m}\right)}^{2}\right]\right\}}^{2}$$

for progressive nucleation [29]. It is evident that for progressive nucleation, a steeper current increase is observed in comparison to the instantaneous case, allowing to discriminate nucleation type. In the same figure we reported the experimental current transient of our sample, as an example, deposited with a cathodic potential of 0.35 V for 3 min. Our experimental transient curve shows a trend compatible with the instantaneous nucleation process. For t > t_m_, the experimental curve results slightly higher than the theoretical one, probably because of a more efficient diffusion process. Similar comparison was done for all experimental current transients, confirming that WO_3_ electrodeposition is characterized by an instantaneous nucleation process, probably ascribed to the ITO substrate in which the surface could drive a fast formation of WO_3_ nuclei. The instantaneous nucleation could also be confirmed by the evidence that the WO_3_ film is composed of 50 nm sized grains (Figure 2a–f), regardless of the cathodic potential or growth time.

**Figure 7:**
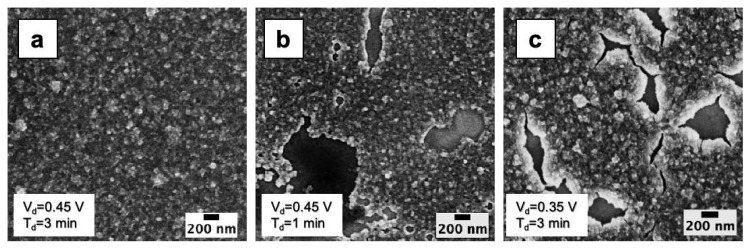
High resolution SEM images of the samples deposited by applying (**a**) 0.45 V for 3 min, (**b**) 0.45 V for 1 min and (**c**) 0.35 V for 3 min.

In order to study the electrodeposition kinetics, it is useful to observe high resolution SEM images (Figure 7) referring to early stage growth. At cathodic potential of 0.45 V, t_m_ and t_s_ are 7 s and 100 s, respectively, thus we found, as expected, a full coverage of substrate only after 100 s (Figure 7a), while at 60 s the electrodeposited WO_3_ (Figure 7b) presents many holes (200 nm sized) waiting to be filled by prolonging the growth. By reducing the cathodic potential to 0.35 V, a depositon of 3 min (Figure 7c) gives a similar result to the 60 s-0.45 V sample, with many holes surrounded by the growing WO_3_. Thus, we can assume that the electrodeposition transient proceeds with the growth of isolated grains up to t_m_, then merging of grains occurs roughly up to t_s_, and an almost continuous film is achieved after t_s_. Such considerations are useful to drive the deposition of a nanostructured versus continuous WO_3_ film, as for times between t_m_ and t_s_, the uncomplete substrate coverage, leading to the observed holes, could increase the surface over volume ratio of W oxide.

### 3.3. Model for WO_3_ Electrodeposition

**Figure 8:**
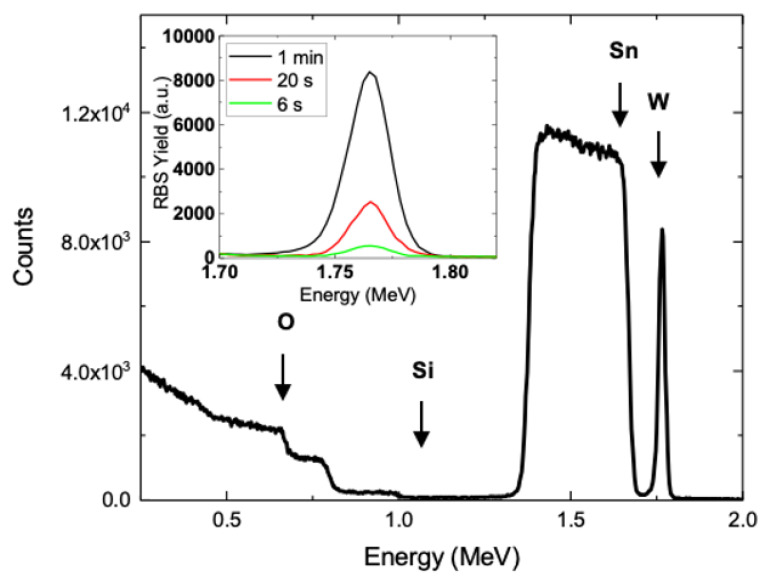
Rutherford backscattering spectrometry (RBS) spectra of electrodeposited WO_3_. The normal configuration is used for the determination of W dose from the integration of the peak related to the W presence for the sample deposited by using V_d_ = 0.45 V for 1 min; Inset: W peak of samples deposited by using V_d_ = 0.45 V for 6 s, 20 s and 1 min.

**Figure 9:**
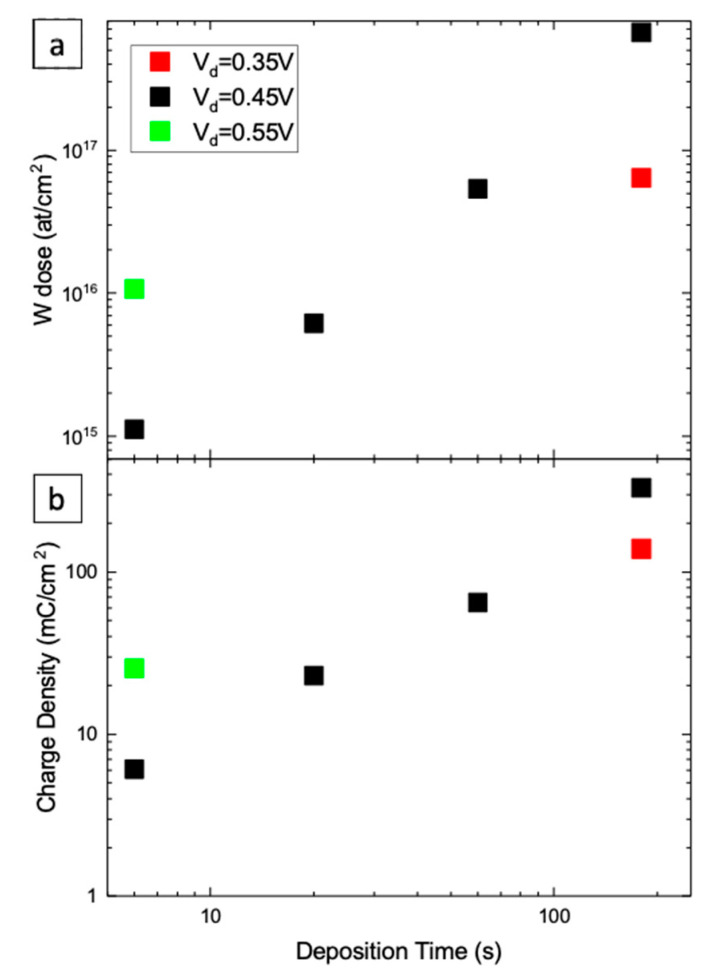
(**a**) W dose obtained from RBS spectra as a function of t_d_ for different V_d_ values; (**b**) charge density obtained from the integration of the current transient as a function of t_d_ for different V_d_ values.

Figure 8 shows the RBS spectra of samples deposited at 0.45 V for 60 s. The arrows indicate the energy of He ions backscattered by W (1.836 MeV), Sn (1.752 MeV), Si (1.138 MeV) or O (731.8 keV) atoms on the surface of analyzed films. The signals for W and O are present as expected, while Sn (present in the ITO) and Si (glass below ITO) ones appear at lower energy since they are buried below WO_3_ and ITO films, respectively. The inset reports only the W signal at increasing deposition times. The area below the W peak quantifies the W dose on the analyzed sample. The result of such exercise is reported in Figure 9a for different deposition parameters. The O content of the WO_3_ film is more challenging because of the overlapping of the RBS signal related to underlying layers (ITO and glass). To overcome this, RBS spectra in the glancing configuration were acquired to enhance backscattering from the surface (Figure S5). A W:O = 1:3 stoichiometry was obtained by comparing the O and W dose in selected samples.

Figure 9a (log-log scale) reports the dose of W in electrodeposited films as a function of t_d_. An increase with time of W dose is visible for 0.45 V. At lower and higher cathodic potential, a corresponding lower and higher W dose is found, as expected. As far as the W deposition rate is concerned, at 6 s an average value of 1.7 × 10^14^ at./cm^2^ s is obtained, while at 180 s, the average rate increases to 4 × 10^15^ at./cm^2^ s. Figure 9b shows the time variation of electrical charge exchanged at working electrode per unit area of WO_3_ film (obtained as time integral of current density). These last data follow the same trend observed in Figure 9a and roughly agree with W dose, suggesting that with increasing current an increasing deposition rate occurs. In order to correlate these data with the growth kinetics, a model for the WO_3_ electrodeposition is needed.

According to Meulenkamp, electrodeposition of WO_3_ from a PTA solution proceeds via a reduction process, starting with PTA dissociation [30]

(3) $$2W+10H_{2}O_{2}\to W_{2}O_{11}^{2-}+2H^{+}+9H_{2}O$$

and followed by reduction of the peroxotungstate ion W_2_O_11_^2−^ towards WO_3_ formation [30]

(4) $$W_{2}O_{11}^{2-}+\left(2+2x\right)H^{+}+2{\mathrm{xe}}^{-}\to 2{\mathrm{WO}}_{3}+\frac{2+2x}{2}H_{2}O+\frac{8-2x}{4}O_{2}$$

In the last reaction, x is the number of electrons exchanged to deposit a single WO_3_ unit, thus relating the current at the working electrode to the deposition rate. According to the above chemical reaction, x = 0 or 4 leads to high or null O_2_ production. A high (low) value of x is related to a low (high) effectiveness of current versus deposition rate, since a large (small) current exchanged at the working electrode is required to proceed with WO_3_ deposition. Meulenkamp experimentally showed that x ranges between 1 and 3.5 [30].

**Figure 10:**
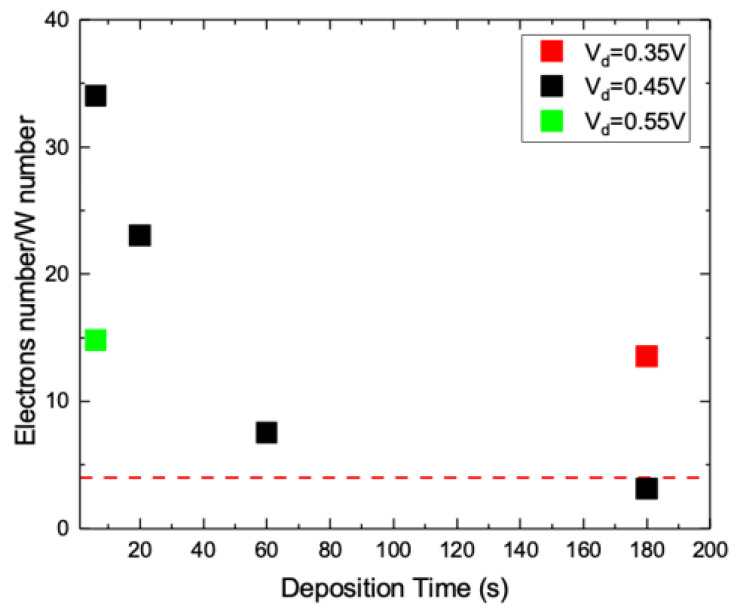
Exchanged electrons during the deposition of a single W atom for the samples deposited at different V_d_ for different t_d_.

Figure 10 reports the x value measured per each sample. This value is the ratio between W dose (Figure 9a) and charge exchanged at the working electrode (Figure 9b). For a cathodic potential of 0.45 V, a decreasing trend for x is clearly visible with time, starting from x = 34 at the beginning, and reaching x = 3.12 only at 180 s, well after t_s_. This reveals that for the whole transient the effectiveness of current versus deposition rate is very low. In fact, at early stage the W deposition rate is quite low in comparison to longer times, but it requires a current higher than at longer times. It is worth noting that x reaches a value which is in accordance with the reaction (2) as soon as the transient ends up, i.e., at t_s_. After the current transient, the growth of WO_3_ film proceeds with an effective use of exchanged charges at the working electrode. In comparison to 0.45 V, higher or lower cathodic potentials leads to lower or higher x values for the same deposition time. This evidence confirms that the effectiveness of current is potential dependent as the current transient is.

After the transient, the WO_3_ deposition seems to be compatible with the following reaction:

(5) $$W_{2}O_{11}^{2-}+10H^{+}+8e^{-}\to 2WO_{3}+5H_{2}O$$

for which no O_2_ production is needed. For longer times, the x value saturates and a constant chemical reaction like the above one can describe the WO_3_ electrodeposition. At the early stage, some auxiliary process must be invoked to account for the larger current observed. Actually, during the transient an evident color change of samples is observed, which can be caused by an H intercalation [1,2], made even more effective by the holes present in the WO_3_ layer (Figure 7). The H intercalation is a reduction process, which can explain the quite larger current and the very high x value observed during the transient.

## 4. Conclusions

The electrodeposition of WO_3_ film from a PTA solution was investigated at different cathodic potentials (0.35–0.65 V) and deposition times (2–1800 s). At long times and/or high cathodic potential, a broken WO_3_ film is obtained. Several combinations of electrodeposition parameters (deposition map) have been used to investigate the film synthesis and its morphology (continuous or broken films). The growth kinetics before breaking were deeply investigated. The electrodeposition of a WO_3_ film proceeds with an initial transient stage, with partial coverage of the substrate and a time-dependent current, followed by a steady state stage leading to continuous film and constant current. The WO_3_ electrodeposition on ITO substrates shows an instantaneous nucleation and is diffusion-controlled, producing nanostructured films (50 nm sized grains) with 1:3 W:O stoichiometry. A quantitative analysis compared the current at the working electrode to the W deposition rate, validating a chemical reaction underlying the microscopic mechanism for WO_3_ deposition at longer times. At the early stages of deposition, a larger current is observed despite a reduced W deposition rate, and a possible H intercalation process is discussed. The reported modeling of the electrodeposition process can be suitable for a controlled synthesis of WO_3_ nanostructures.

## Supplementary Materials

The following are available online at https://www.mdpi.com/2079-4991/10/8/1493/s1, Figure S1: (a) AFM image of the sample deposited by applying V_d_ = 0.45 V for 6 s. The blue line identifies the region in which the line spectrum showed in (b) is obtained, Figure S2: Tauc plot of sample deposited at V_d_ = 0.45 V for 3 min. The red line is the linear fit, Figure S3: XRD pattern of the sample deposited by applying V_d_ = 0.45 V for 3 min. The peaks are related to the ITO presence in the substrate, Figure S4: Current transients recorded during the samples electrodeposition by applying different V_d_ for 3 min. The dotted lines allow to individuate the different t_s_ for the different V_d_ values, Figure S5: RBS spectrum, of the sample deposited by using V_d_ = 0.45 V for 1 min, acquired in glancing configuration. The red line is the line of the background, which was subtracted in order to individuate the peak related to the O content (green line).
