# Supplementary material for: Investigation of WO3 Electrodeposition Leading to Nanostructured Thin Films
Source: Nanomaterials (Basel). 2020 Jul 30;10(8):1493. doi: 10.3390/nano10081493 (PMC7466470; doi:10.3390/nano10081493)
Supplement: Supplementary file 1 [file nanomaterials-10-01493-s001.pdf]

nanomaterials

Article

Investigation of WO_3 Electrodeposition Leading to Nanostructured Thin Films

G. Mineo *, F. Ruffino, S. Mirabella, E. Bruno

Dipartimento di Fisica e Astronomia "Ettore Majorana",Università di Catania, and CNR-IMM, via S. Sofia 64, 95123 Catania, Italy; francesco.ruffino@dfa.unict.it (F.R.); salvo.mirabella@dfa.unict.it (S.M.); elena.bruno@dfa.unict.it (E.B.)

* Correspondence: giacometta.mineo@dfa.unict.it

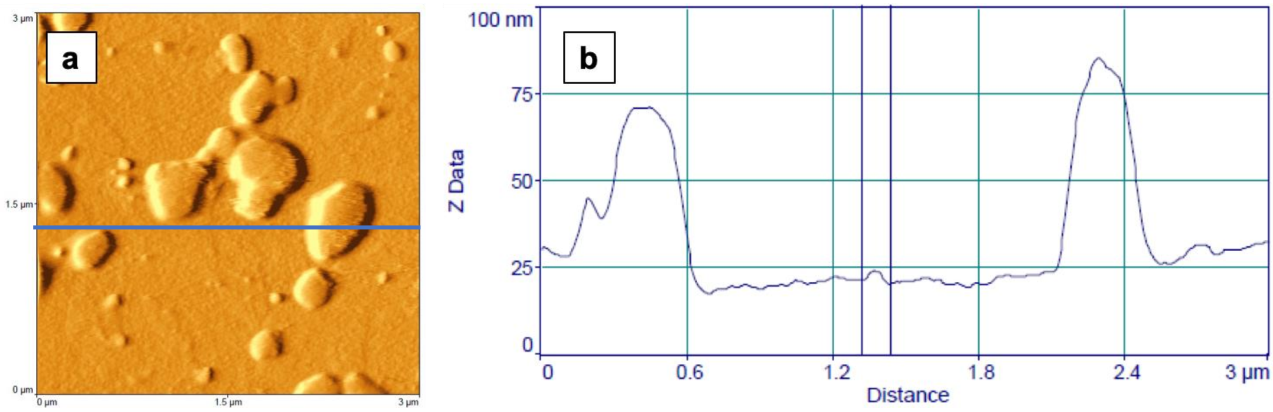

Figure S1. (**a**) AFM image of the sample deposited by applying V_d = 0.45 V for 6 s. The blue line identifies the region in which the line spectrum showed in (**b**) is obtained.

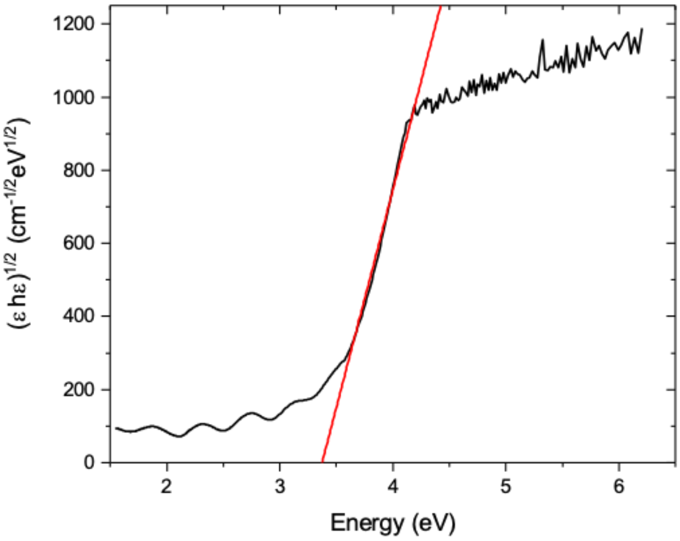

Figure S2. Tauc plot of sample deposited at V_d = 0.45 V for 3 min. The red line is the linear fit.

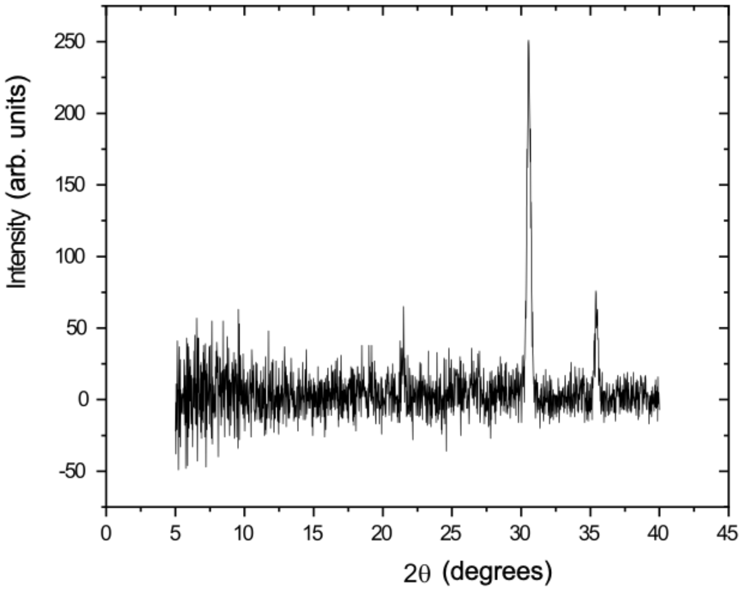

Figure S3. XRD pattern of the sample deposited by applying V_d = 0.45 V for 3 min. The peaks are related to the ITO presence in the substrate.

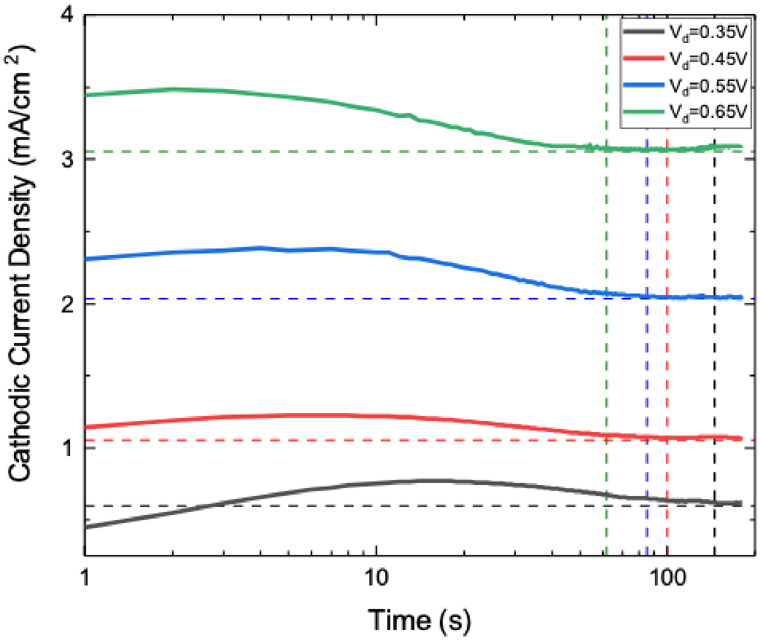

Figure S4. Current transients recorded during the samples electrodeposition by applying different V_d for 3 min. The dotted lines allow to individuate the different t_s for the different V_d values.

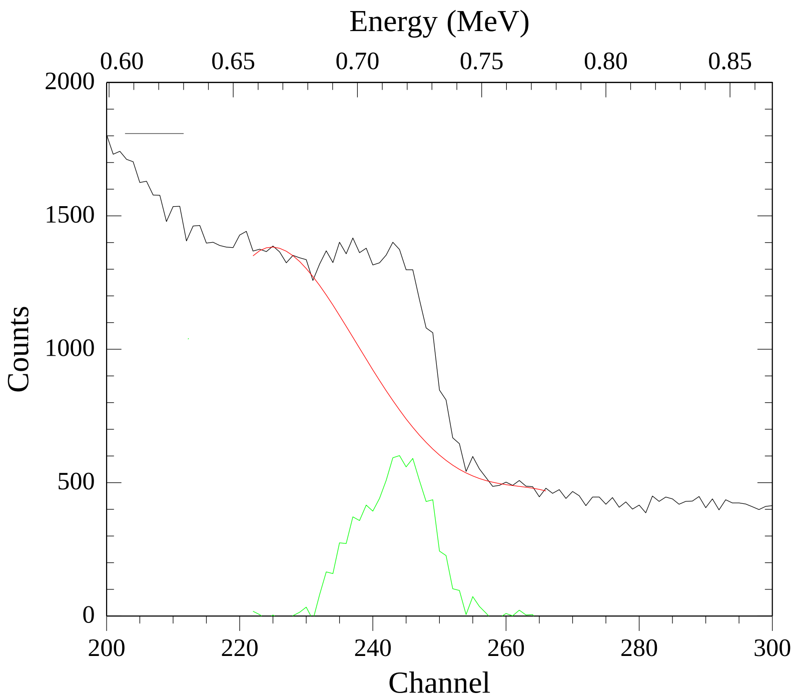

Figure S5. RBS spectrum, of the sample deposited by using V_d = 0.45 V for 1 min, acquired in glancing configuration. The red line is the line of the background, which was subtracted in order to individuate the peak related to the O content (green line).

© 2020 by the authors. Licensee MDPI, Basel, Switzerland. This article is an open access article distributed under the terms and conditions of the Creative Commons Attribution (CC BY) license (http://creativecommons.org/licenses/by/4.0/).
